# A Case of Disease, Supposed to Arise from Poison, Attended with Extraordinary Symptoms

**Published:** 1810-11

**Authors:** 

**Affiliations:** Secretary to the Medical Society of Orange County


### *A Case of Disease, supposed to arise from Poison, attended with extraordinary Symptoms. By* Dr. Arnell, *Secretary to the Medical Society of Orange County.*

LITTLETON, a servant man, belonging to Mr. William Phillips, aged 26 years, generally of a healthy appearance through life, began to complain in the month of September, 1806, at distant intervals, of slight nausea, and sometimes vomiting, but so trifling, as not to excite much attention. In October, the aid of a Physician was requested, who found him labouring under the above symptoms, increased, and accompanied with slight strabismus, but without any visible dilation of the pupil, and a slight obtuse pain in the head, attended with drowsiness and lassitude of the system, pulse natural, appetite somewhat impaired, bowels regular, sleep a little disturbed. The general course of prescriptions through this month were, 1st, Emetics, without relief; 2d, Cathartics, and 3d, blood letting, equally unsuccessful. In the latter part of this month some of the symptoms became materially increased, to wit, nausea with frequent disposition to vomit, strabismus, cardialgia, appetite bad, a slight disposition to diarrhœa with tenesmus, sleep disturbed with frightful dreams, urine a little turbid, pulse natural, pupils of the eye not visibly dilated. Prescriptions during this period were, cathartics, of different kinds, blood letting, and epispastics behind the ears; all without any visible effect for the better. In the fore part of November the foregoing symptoms became materially increased; an almost constant retching to vomit, tenesmus, and diarrhœa extremely distressing though little discharged, slept very little, started often, screaming as if terrified, strabismus much increased, pulse regular, epistaxis frequent, blood buffy, appetite entirely gone, obtuse pain in the head. The predominating symptoms agreeing so well with those describ'd by Dr. Quin, on Hydrocephalus internus, led to a conclusion that water was collecting in the brain. The patient was strongly impressed that some kind of poison had been given him by a female servant, and in consultation with three gentlemen of the faculty, we became divided in sentiment; it was therefore considered adviseable, in some measure, to alter the prescriptions, and consider poison, some time past administered, as constituting the cause of his disease. Epistaxis more frequently occurring, recourse was had to more frequent blood letting, but without checking it, except for a few minutes, and though he was often bled until near fainting, the pulse became but little weaker, and that for a short time. Alka-

Alkalines, in small doses, were often given, but without effect, and the warm bath made use of with as little success. At this period the pulse was about 80, and rather tense, but the strength of the patient appeared rapidly failing; the disposition to vomit became less with an increase of tenesmus, pain of the head somewhat diminished. About the 10th of November the loss of sight was almost total, sleep much disturbed, epistaxis almost constant, strength rapidly failing, pulse rather weaker than in health, number about 80; cardialgia, attended with extreme anxiety and uneasiness of the whole body; these constituted the most prominent symptoms during the last days of his illness. Dr. Phillips, who was present during the last moments of life, bled him twice on the last day; the blood showed an inflammatory crust. The epistaxis continued from its commencement till his death; blood letting once and twice a day was resorted to, as the strength of the patient would admit; the pulse continued regular, and about 80 strokes a minute, till within fifteen minutes of his decease.

From the extraordinary and peculiar circumstances of the above complaint, it is to be regretted that an exact statement of the symptoms and treatment had not been made from its commencement; but the disease assumed a more formidable and interesting appearance than was at first contemplated.

His master being in New York when he died, I could not obtain liberty to dissect him until his return, which was 83 hours after his decease; when the following were the appearances upon dissection.

No external appearance to characterize any disease; no putrid effluvia, no purple or livid spots, no unusual appearance of the genitals; the epidermis was close and firm, the hair firmly fixed in the scalp, abdomen not in the least tumid, but perfectly collapsed as when he first died. On dividing the integuments of the cranium they appeared white, sound, and perfectly natural. On removing the integuments and the upper part of the cranium, nearly horizontally, the blood-vessels of the brain appeared very turgid, and exhibited strong marks of inflammation; two small vesicles of water appeared on the superior lobe of the left hemisphere of the brain, just beneath the pia mater. After dividing the two hemispheres through the falx, and corpus callosum, down to the cerebellum, two transverse incisions were made through the cerebrum horizontally, so as completely to expose the right and left ventricles; there was no collection of serum or any other fluid. On laying open the abdomen, the intestines appeared sphacelated in detached portions through their whole extent. The mesentery was quite putrid, but the integuments and muscles appeared entirely sound, shewing no signs of gangrene. The stomach stomach was quite empty, its external appearance quite uniform shewing no more signs of decomposition than might be expected, considering the time since his death. On opening the stomach, the internal coat appeared to have considerably suffered from inflammation, was uniformly sphacelated, and very much so in comparison with the external or muscular coats.

If I had not been disappointed in finding water in the ventricles of the brain, my impressions would have remained, that his case was hydrocephalus internus; but as it was, the symptoms and appearances, upon dissection, do not agree with those which take place after taking poison, nor any other disease marked by nosologists. I am perfectly at a loss where to rank it, but am convinced that it depended upon too great excitement of the system, and am satisfied with the treatment. It may not be improper to remark, that I have, in two instances, the last summer, cured the epilepsy with the acetate of lead and sal: martis.

